# In Elderly Patients With Antipsychotic-Induced Hyperprolactinemia, Could Switching to a Prolactin-Sparing Antipsychotic or Adding a Dopamine Agonist, Rather Than Maintaining the Current Regimen, Normalize the Prolactin Levels Without Triggering Psychotic Relapse or Increasing the Risk of Life-Threatening Adverse Events? A Systematic Review

**DOI:** 10.1192/bjo.2025.10236

**Published:** 2025-06-20

**Authors:** Gaurav Uppal, Sathyan Soundara Rajan, Akhila Bhandarkar, Sneh Babhulkar, Asha Devi Dhandapani

**Affiliations:** 1 Satyam Hospital, Ludhiana, India; 2 BCUHB, Wrexham, United Kingdom; 3 KSHEMA, Mangalore, India; 4 Greater Glasgow and Clyde NHS Trust, Glasgow, United Kingdom

## Abstract

**Aims:** Elevated prolactin levels due to antipsychotic drugs are prevalent in elderly patients and may cause multiple complications. Hence, the purpose of the present study is to compare the effectiveness of changing existing antipsychotic treatments with prolactin-sparing antipsychotics or adding dopaminergic agents to the existing treatment in patients with clinically insignificant hyperprolactinaemia in later life.

**Methods:** The sample for this systematic review was identified using a broad search strategy in key electronic databases including Pubmed, SIGLE, CINAHL, Web of Science and OVID. To complete the search, only citations that included elderly or geriatric patient populations and hyperprolactinemia associated with antipsychotic medications were used. Normalization of prolactin levels, psychiatric status, and side effects were the main results measured.

**Results:** The review flagged several main studies: The efficacy of antipsychotic aripiprazole use in the treatment of schizophrenia is discussed about its impacts on prolactin levels in individuals of different ages and gender. There was no effect on prolactin plasma concentrations in postmenopausal patients with depression and a small but significant positive impact in schizophrenia patients.

An innovation that supplements the pattern of traditional Chinese medicine together with a low dose of aripiprazole can be useful for treating antipsychotic-induced amenorrhea. Prolonged exposure to prolactin-elevating antipsychotics was found to raise the risk of fractures, a finding that provided insight into other health risks.

**Conclusion:** The approach to the management of antipsychotic-induced hyperprolactinemia in older adults is beyond general management. Although studies that counter the aversive effects of antipsychotics with drugs like aripiprazole seem promising, its benefits are somehow relative across populations. Since there may be long-term health risks such as fractures in the future, it is taken fairly seriously and requires vigilance with a concrete individual management plan.

Regarding the limitation of the present study, it is recommended that future research incorporates different antipsychotics, follow-up outcomes longer, and provide strategies to avoid such risk factors among this population.

